# Response of a Coastal Groundwater System to Natural and Anthropogenic Factors: Case Study on East Coast of Laizhou Bay, China

**DOI:** 10.3390/ijerph17145204

**Published:** 2020-07-18

**Authors:** Ya Sun, Shiguo Xu, Qin Wang, Suduan Hu, Guoshuai Qin, Huijuan Yu

**Affiliations:** School of Hydraulic Engineering, Dalian University of Technology, Dalian 116024, China; yasun_dlut@163.com (Y.S.); simplewang@mail.dlut.edu.cn (Q.W.); husuduan@mail.dlut.edu.cn (S.H.); qgs1991@mail.dlut.edu.cn (G.Q.); yuhuijuan@mail.dlut.edu.cn (H.Y.)

**Keywords:** coastal area, groundwater level, groundwater quality, response, changing environment

## Abstract

With a shifting climate pattern and enhancement of human activities, coastal areas are exposed to threats of groundwater environmental issues. This work takes the eastern coast of Laizhou Bay as a research area to study the response of a coastal groundwater system to natural and human impacts with a combination of statistical, hydrogeochemical, and fuzzy classification methods. First, the groundwater level dynamics from 1980 to 2017 were analyzed. The average annual groundwater level dropped 13.16 m with a descent rate of 0.379 m/a. The main external environmental factors that affected the groundwater level were extracted, including natural factors (rainfall and temperature), as well as human activities (irrigated area, water-saving irrigated area, sown area of high-water-consumption crops, etc.). Back-propagation artificial neural network was used to model the response of groundwater level to the above driving factors, and sensitivity analysis was conducted to measure the extent of impact of these factors on groundwater level. The results verified that human factors including irrigated area and water-saving irrigated area were the most important influencing factors on groundwater level dynamics, followed by annual precipitation. Further, groundwater samples were collected over the study area to analyze the groundwater hydrogeochemical signatures. With the hydrochemical diagrams and ion ratios, the formation of groundwater, the sources of groundwater components, and the main hydrogeochemical processes controlling the groundwater evolution were discussed to understand the natural background of groundwater environment. The fuzzy C-means clustering method was adopted to classify the groundwater samples into four clusters based on their hydrochemical characteristics to reveal the spatial variation of groundwater quality in the research area. Each cluster was spatially continuous, and there were great differences in groundwater hydrochemical and pollution characteristics between different clusters. The natural and human factors resulted in this difference were discussed based on the natural background of the groundwater environment, and the types and intensity of human activity.

## 1. Introduction

The coastal areas are resource-rich and have favorable natural conditions, supporting high population density and intensive anthropological activities. However, the groundwater environment in coastal regions is considered to be particularly vulnerable and sensitive to changing environments [1,2,3,4,5,6]. Changing environment mainly refers to climate change and human activities. Climate change alters regional hydrological features and environmental factors, which in turn affects water circulation, hydrodynamic conditions as well as the source, distribution and migration of pollutants [7]. Human activities, such as large-scale development and utilization of surface and ground water, water conservancy projects, and urbanization have changed the groundwater dynamics and hydrochemical field in a more direct and immediate way [8,9,10]. With the continuous social and economic growth, the magnitude, spatial scale, and pace of human-related change in coastal groundwater system has far exceeded that caused by natural evolution. Due to shifting weather pattern and the enhancement of human activities, coastal areas are exposed to threats of groundwater environmental issues such as decreasing groundwater levels, seawater intrusion, groundwater pollution, and soil salinization [11,12]. Understanding the magnitude of impact associated with natural and human factors on coastal groundwater system, and process and mechanism of groundwater environment evolution, can provide technical support for coastal groundwater environmental protection and rehabilitation.

Over the last few decades, the impacts of climate change and human activities on groundwater system have attracted extensive attention from researchers. However, the diversity of its engagements, and the highly non-linear relationship with groundwater environment response, render the role of climate change and human activities difficult to assess [13,14]. Therefore, quantitative research should be intensified to facilitate intuitive understanding of their impacts on groundwater system. Some researchers have conducted quantitative research on the effects of natural and human factors on groundwater circulation. This research mainly falls within one or the other of the following: (1) use numerical groundwater flow models to conduct sensitivity analysis of groundwater level dynamics to the driving factors. Chen, H.R. et al. [15] employed Modflow to simulate the response of phreatic water level in Wuqiao, North China Plain under different combinations of weather pattern and intensity of human activities. Hydrological models have clear physical meaning, and can accurately describe the groundwater circulation. While the disadvantages lie in the high-volume data required for modeling, and complexity of model calibration and verification. (2) Use statistical methods to measure the impacts of influencing factors. Wang, J.Z. et al. [16] used comprehensive index method to evaluate the impacts of human activities on shallow groundwater in different eras in Hutuo River Plain, China. The results showed that the disturbance from human activity have progressively increased. Feng, S.Y. et al. [17] developed a Back-propagation Artificial Neural Network (BP-ANN) model to simulate the dynamic variation of groundwater table in an agriculture-based area of Northwest China. The model quantitatively illustrated the influence of natural & human factors on groundwater level with sufficient high accuracy. The sensitivity analysis demonstrated that human activities and reduction of surface water inflow were the main causes leading to groundwater-level decline. Liu, J. et al. [18] adopted principal component analysis (PCA) method to determine the main factors driving change in groundwater depth in an irrigated zone. The results revealed that compared with natural elements, human activities including groundwater extraction and surface water irrigation consumption played a leading role in groundwater depth. Statistical methods exam the statistical relationship between the variables to reflect their implicit intrinsic linkages without consideration of physical meaning; thus, provide great simplicity and flexibility over numerical models.

The particularly useful and universal approach to assess human-caused impacts on groundwater quality is to evaluate the changes of groundwater chemical indicators based on mathematical statistical methods. Groundwater chemical abnormality can potentially be used to reflect the pressure applied to the groundwater system, as the increased volatility and dispersion of groundwater components under the influence of human activities [19]. However, the selection of the indicators, and the weighting and scoring system are not immune to subjectivity. Zhao, W. et al. [20] used variation coefficient method to calculate the weight of groundwater chemical indicators. Liu, L. et al. [21] analyzed the sensitivity of groundwater quality indicators and their relevance with descriptive, cluster, and principal component analysis for screening representative indicators. Nevertheless, it was argued that the statistical method measures human impacts based on the degree of variation of limited indicators. This raises the possibility of masking and swamping in outlier detection, or erroneous estimation of abnormal intensity, which finally results in imprecise outcome of evaluation [22,23]. The deterioration of groundwater quality is attributed to the combined effects of natural and human factors. In addition to human-related impacts, geological factors may cause poor-quality groundwater. However, large-scale human activities make it difficult to determine natural background levels for groundwater. In that context, the evaluation result of human-made groundwater pollution is often exaggerated. A current research focus is on distinguishing the impacts of anthropogenic and natural factors on groundwater environment, and identifying the extent to which they affect groundwater environment. With the help of multivariate statistics, geostatistics, and GIS technology, Güler, C. et al. [24] characterized the physico-chemical properties of groundwater, identified the major hydrogeochemical processes, and assessed the impact of human activities on groundwater hydrology and hydro-chemistry occurring in the Tarsus costal area. Birnk, C.V.D. et al. [25] provided a quantitative insight into the impacts of land-use pattern on groundwater components in the Netherlands with statistical tools. The results demonstrated that the dominant processes controlling components in groundwater was scale-dependent, with geochemical processes playing a leading role on the national scale while land use pattern on local and regional scales. Re, V. et al. [26] confirmed the strong dependency of groundwater composition on irrigation activities in Bou-Areg coastal aquifer by using coupled multi-tracer and statistical analysis. Water-rock interaction dominated groundwater composition during low irrigation season, while agricultural return flow caused groundwater salinization in high irrigation season naturally. Merchan, D. et al. [27] studied groundwater salinization in Lerma irrigated basin through geochemical modeling and multi-variate statistical analyses. The result pointed to the natural control on salinization process but without visible influence of human activities. Peng, C. et al. [22] and Zhang, X.W. et al. [23] presented that the combination of mathematical statistics and hydrochemical diagrams could theoretically identify the statistical outliers as well as the data of abnormal chemistry characteristics as hydrochemical diagrams reflected the relationship between groundwater components, as well as hydrogeochemical processes controlling groundwater evolution.

This work takes the east coast of Laizhou Bay as an example to study the impacts of natural and human driving factors on coastal groundwater environment. This area is a good example of a coastal aquifer continuously exposed to negative external factors of climate change and intense human activities, which have collectively contributed to the groundwater environmental degradation. In this work, the dynamic change of groundwater level in the study area from 1980 to 2017 was analyzed. The main external environmental factors that affected the groundwater level were extracted, including natural factors (rainfall and temperature), as well as human activities (irrigated area, water-saving irrigated area, sown area of high-water-consumption crops, gross industrial output value index and reservoir water supply). A backpropagation neural network model was built to simulate the response of groundwater level to the above influencing factors. Further, sensitivity analysis was conducted to measure the impact of the influencing factors on groundwater level dynamics. Surface and ground water samples were collected to analyze the physical and chemical characteristics. The formation of groundwater, and the main hydrogeochemical processes controlling the groundwater compositions were discussed based on hydrochemical diagrams and ion ratios to understand the natural background of groundwater environment. The fuzzy C-means clustering was adopted to classify groundwater samples according to the hydrochemical characteristics, to reveal the spatial variability of groundwater quality and identify the main natural factors and human activities that affected groundwater quality.

## 2. Overview of the Study Area 

**Figure 1:**
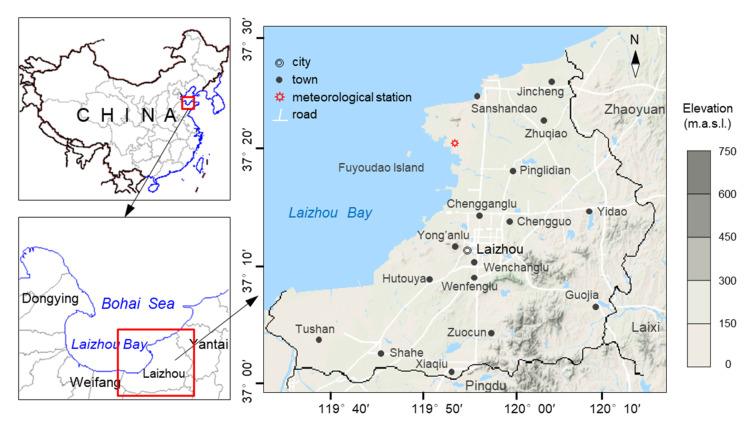
Location and elevation map of study area.

**Figure 2:**
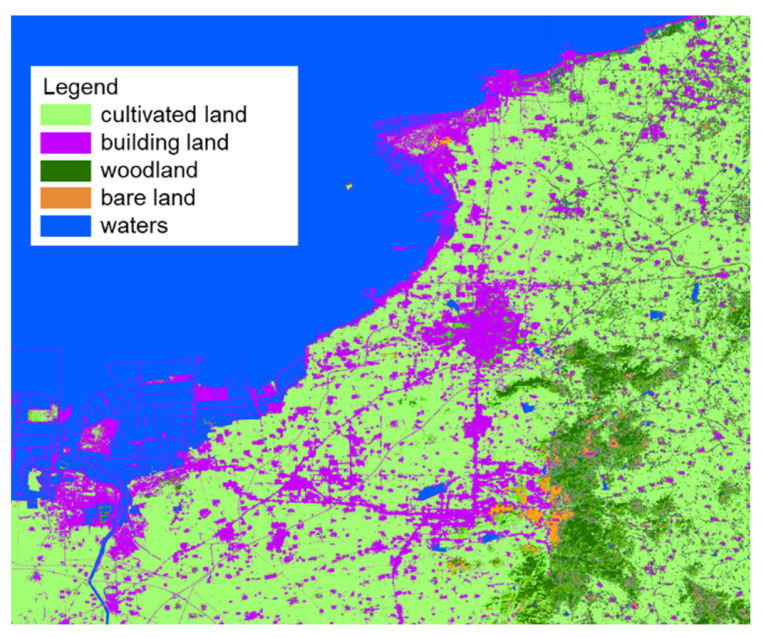
Land use pattern of the study area.

The study is located on the east coast of Laizhou Bay, covering an area of approximately 2000 km^2^ (Figure 1). It has a temperate continental monsoon climate, characterized by four distinct seasons. The temperature averages about 12.5 °C annually. The mean annual precipitation and evaporation are about 580 mm and 1944 mm, respectively (data from Yantai Hydrographic Bureau, China). Rainfall varies dramatically from year to year, and is concentrated from June to September. The terrain slopes from southeast to northwest. The main landforms are low mountains, hills, and plains. Figure 2 illustrates the land use patterns in the study area (data year 2017, spatial resolution 30 m, supported by National Earth System Science Data Center, National Science & Technology Infrastructure of China). Agriculture is the dominant land use category within the area, followed by building land and forest. The offshore region has been substantially reclaimed for marine aquaculture.

**Figure 3:**
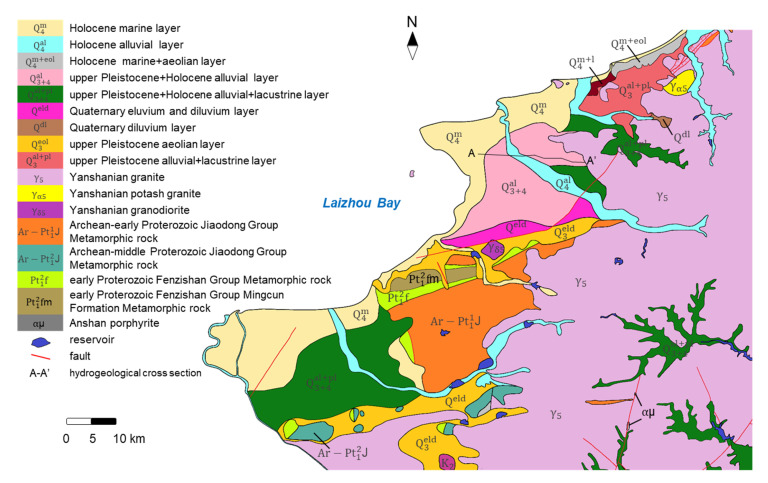
Geological map of the study area.

**Figure 4:**
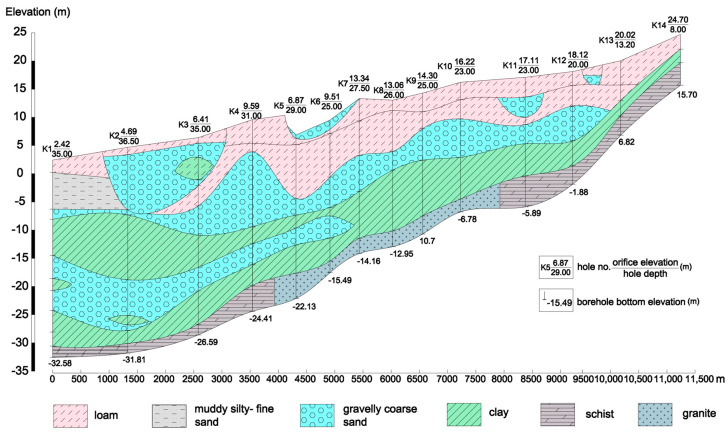
Hydrogeological cross-section (A-A’ profile in Figure 3).

As Figure 3 (data from Yantai Land and Resources Bureau) shows, the strata in the study area are mainly: (1) Proterozoic Lower Jiaodong Group Metamorphic rocks. The dominant lithologies are leptynite interlaced with plagioclase amphibolite, leptynite, and gneiss interlaced with plagioclase amphibolite; (2) Proterozoic Lower Fenzishan Group Metamorphic rocks. The main lithologies include dolomite and tremolite containing talc and magnesite, feldspar quartzite, schist interlaced with marble and leptynite; (3) Proterozoic magmatic rocks. They are widely distributed in the east and south of the study area, and the main lithologies are granite and granodiorite; (4) Tertiary strata. The main lithology is argillaceous sandstone; (5) Quaternary strata, which are extensively distributed in coastal plains and river terraces. The faults host large accumulation of gold and iron deposits. Figure 4 displays a hydrological cross section in the study area. 

Most of the rivers rise in southeastern hilly areas and flow towards the Bohai Sea. They are seasonal rivers mainly fed by monsoon. In recent years, due to below-normal levels of precipitation and upstream reservoir water impoundment, river runoff of has been greatly reduced.

There are two main types of aquifers in the research area: unconsolidated deposit aquifer, which is mainly distributed in river valleys, alluvial plains, and coastal plains; and bedrock aquifer created by the weathering fissures and structural fractures of metamorphic rocks and granites in the hilly areas. The groundwater flow follows the topography. Groundwater is replenished largely by precipitation and to a smaller extent by surface water (irrigation return flow, river, water reservoir, etc.), and is discharged mainly by artificial exploitation for agricultural, industrial, and domestic use. Since the 1970s, there is a substantial increase in water demand owing to the sustained economic growth. Due to serious surface water resources constraints, water supply in the study area has long relied on groundwater on account of its ubiquitous occurrence, easy availability, and reliability. The extensive groundwater over-exploitation under weak management and planning led to sharp decline in local groundwater levels, which triggered the aggressive landward movement of salt water. The degradation of groundwater environment posed serious threats to local industry and agriculture development, as well as people’s health.

## 3. Materials and Methods

### 3.1. Water Sample Collection and Analysis

**Figure 5:**
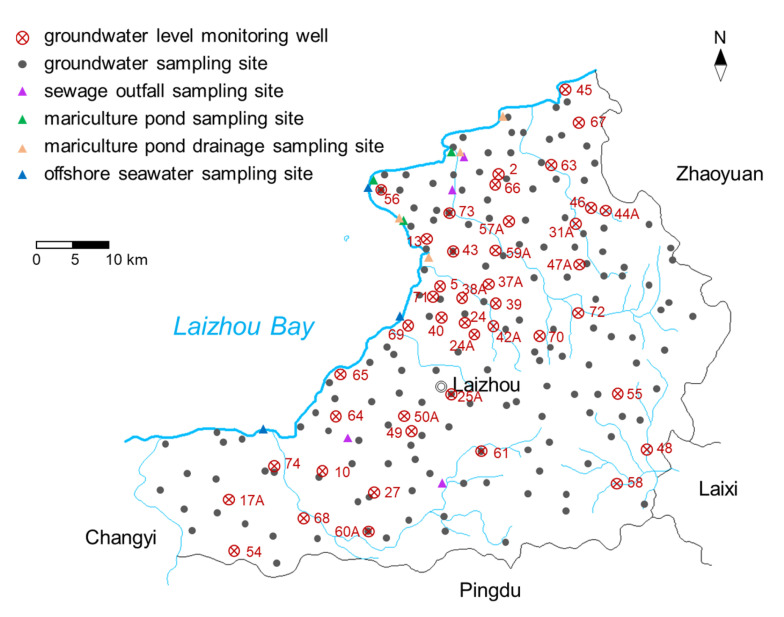
Location map of surface water and groundwater sampling sites.

A total of 153 groundwater and 14 surface water samples were collected in the research area from April to May, 2017. The location of the sampling sites is shown in Figure 5. The layout of groundwater sampling points, as well as collection, storage, and transportation of groundwater samples are performed under the guidance of the Technical Specifications for Groundwater Environmental Monitoring (HJ/T 164-2004). Ministry of Ecology and Environment of the People’s Republic of China, China Environmental Science Press, 2004. Groundwater samples were collected from local agricultural irrigation wells, rural household hand pumps, and groundwater level monitoring wells. Surface water samples were collected from factory sewage outfalls, aquaculture ponds, drainage ditches on aquaculture ponds and offshore waters. Replicate samples were collected for 20% of the water samples. All the samples were stored in plastic or glass bottles rinsed with deionized water. After adding into fixatives and sealed, the samples were refrigerated at 4 °C (with exclusion of light) for laboratory analysis. The temperature, pH, salinity, conductivity (EC), and dissolved oxygen (DO) of water samples were detected by the Multi 340i water quality analyzer (WTW, Munich, Germany) on site. Each sampling point was GPS-located and information including water use, groundwater level, land use, etc. were recorded. The selected groundwater quality indicators included the most abundant ions in groundwater (Ca^2+^, Mg^2+^, Na^+^+K^+^, ${SO}_{4}^{2-}$, Cl^−^, ${HCO}_{3}^{-}$ +${CO}_{3}^{2-}$), ${NO}_{3}^{-}$-N, ${NH}_{4}^{+}$-N, ${NO}_{2}^{-}$-N, total dissolved solids (TDS), COD_Mn_, total coliforms, volatile phenols, heavy metals (Cr^6+^, Pb, Cu, Zn) and cyanide. Surface water quality indicators included ${NO}_{3}^{-}$-N, ${NH}_{4}^{+}$-N, ${NO}_{2}^{-}$-N, total nitrogen (TN), COD_Mn_, volatile phenol, Cr^6+^, Pb and cyanide. The determination of the above indicators was carried out in accordance with *Water and Wastewater Monitoring and Analysis Methods (4th Edition), State Environmental Protection Administration of China, China Environmental Science Press, 2012*. Na^+^, K^+^, Ca^2+^, Mg^2+^, Pb, Cu, and Zn were determined by flame atomic absorption spectrophotometry; ${HCO}_{3}^{-}$ and ${CO}_{3}^{2-}$ were determined by acid-base titration; ${SO}_{4}^{2-}$, Cl^−^, ${NO}_{3}^{-}$-N, and ${NO}_{2}^{-}$-N were determined by ion chromatography; ${NH}_{4}^{+}$-N was determined by colorimetric method with Nessler’s reagent; TN was determined by potassium persulfate oxidation-ultraviolet spectrophotometry method; TDS was determined by gravimetric method; COD_Mn_ was determined by spectrophotometric method using sodium oxalate solution; total coliform was determined by membrane filtration method; volatile phenols were determined by 4-aminoantipyrine spectrophotometry method; Cr^6+^ was determined by diphenyl carbonyl dihydrazine spectrophotometry. Cyanide was determined by isonicotinic acid-barbituric acid spectrophotometry.

### 3.2. BP-ANN Model

#### 3.2.1. Basic Theory and Algorithm

The BP-ANN was employed for modeling groundwater level dynamics to quantitatively describe the influence of natural and human factors on groundwater level in the study area. BP-ANN is a kind of multi-layer feedforward network, which is trained by the error back-propagation algorithm. Back-propagation trains the network through the chain rule. After each forward pass through the network, back-propagation performs a backward pass while fine-tuning the parameters of the model (connection weights and biases) based on the error rate obtained in the previous learning epoch. With excellent ability of nonlinear mapping (without the need to reveal mathematical equations describing the mapping relationship in advance), it is one of the most commonly used neural networks. The BP-ANN model has a simple structure, usually composed of 1 input layer, 1 output layer, and 1 or more hidden layers. Numerous studies have suggested that a 3-layer neural network is able to approximate almost any nonlinear mapping functions with any accuracy, provided a sufficient number of hidden neurons [28]. BP network has been used for hydrological simulation, such as flood forecasting, rainfall-runoff simulation and groundwater level dynamics simulation [29,30,31,32]. In this paper, the BP-ANN model is targeted at simulating the variation of groundwater level in response to climate change and anthropological activities. This issue falls within the scope of groundwater hydrological simulation. Therefore, a 3-layer BP network can satisfactorily address the function mapping and fitting needs [31,32].

For a 3-layer BP network, assume that the number of neurons in the input layer, hidden layer and output layer are n, p, and q, and there are m input-output examples for training the network. Define the following symbols:

input vector $X=\left(x_{1},\;x_{2},\;\ldots ,x_{n}\right)$ and expected output vector $Y=\left(y_{1},\;y_{2},\;\ldots ,y_{q}\right)$;

input vector of the hidden layer $Hi=\left(hi_{1},\;hi_{2},\;\ldots ,hi_{p}\right)$, output vector of the hidden layer $Ho=\left(ho_{1},\;ho_{2},\;\ldots ,ho_{p}\right)$, input vector of the output layer $Yi=\left(yi_{1},\;yi_{2},\;\ldots ,yi_{q}\right)$, output vector of the output layer $Yo=\left(yo_{1},\;yo_{2},\;\ldots ,yo_{q}\right)$; 

the connection weight matrix between the input layer and the hidden layer $w_{ij},i=1,2,\ldots ,n,\;j=1,2,\ldots ,p$, and the bias vector for the neurons in the hidden layer $\theta h_{j},\;j=1,2,\ldots ,p$. the connection weight matrix between the hidden layer and the output layer $v_{jt},\;j=1,2,\ldots ,p,\;t=1,2,\ldots ,q$, and the bias for vector the neurons in the output layer $\theta o_{t},\;t=1,2,\ldots ,q$; transfer function f(·), error function e, stop threshold ε and maximum number of training epochs M.

The steps of the algorithm are as follows:

1. assign initial values randomly selected from [−1, 1] for the connection weights $w_{ij}$, $v_{jt}$, the biases $\theta h_{j}\;\mathrm{and}\;\theta o_{t}$. Set the error function e, stop threshold ε and maximum number of training epochs M;

2. select randomly the k_th_ input-output example from the m training samples$:\;X_{k}=\left(x_{1}^{k},x_{2}^{k},\;,\;\ldots ,x_{n}^{k}\right)$, $Y_{k}=\left(y_{1}^{k},y_{2}^{k},\;,\;\ldots ,y_{q}^{k}\right)$;

3. calculate the input of each neuron in the hidden layer $hi_{j}^{k}$ with input $X_{k}$, input-hidden layer connection weight $w_{ij}$ and hidden layer bias $\theta h_{j}$, and then calculate the output of each neuron in the hidden layer $ho_{j}^{k}$ with the transfer function. Similarly, calculate the input of each neuron in the output layer $yi_{t}^{k}$ and the output $yo_{t}^{k}$ with equation (1):

(1) $$hi_{j}^{k}=\overset{n}{\underset{i=1}{\sum }}w_{ij}x_{i}^{k}-\theta h_{j},\;ho_{j}^{k}=f\left(ho_{j}^{k}\right),\;j=1,2,\ldots ,p\;yi_{t}^{k}=\overset{p}{\underset{j=1}{\sum }}v_{jt}ho_{j}^{k}-\theta o_{t},\;yo_{t}^{k}=f\left(yi_{t}^{k}\right),\;t=1,2,\ldots ,q$$

4. calculate the generalized error $eo_{t}^{k}$ for each neuron in the output layer based on the expected output and actual output. Similarly, calculate the generalization error $eh_{j}^{k}$ of each neuron in the hidden layer with $v_{jt}$, $eo_{t}^{k}$ and $yo_{t}^{k}$ with Equation (2):

(2) $$eo_{t}^{k}=\left(y_{t}^{k}-yo_{t}^{k}\right)\cdot yo_{t}^{k}\cdot \left(yo_{t}^{k}-yo_{t}^{k}\right)\;eh_{j}^{k}=\left[\overset{q}{\underset{t=1}{\sum }}eo_{t}^{k}\cdot v_{jt}\right]\cdot yo_{t}^{k}\cdot \left(1-yo_{t}^{k}\right)$$

5. correct the hidden-output layer connection weight and output layer bias using the $eo_{t}^{k}$ and $ho_{j}^{k}$. Similarly, correct the input-hidden layer connection weight and hidden layer bias using $eh_{j}^{k}$ and $x_{i}^{k}$ with Equation (3):

(3) $$v_{jt}\left(N+1\right)=v_{jt}\left(N\right)+\alpha \cdot eo_{t}^{k}\cdot ho_{j}^{k},\theta o_{t}\left(N+1\right)=\theta o_{t}\left(N\right)+\alpha \cdot eo_{t}^{k}\;w_{ij}\left(N+1\right)=w_{ij}\left(N\right)+\beta \cdot eh_{j}^{k}\cdot x_{i}^{k},\;\theta o_{t}\left(N+1\right)=\theta o_{t}\left(N\right)+\beta \cdot eh_{j}^{k}$$

Where, α and β are learning efficiencies, 0 < α < 1, 0 < β < 1.

6. randomly select the next learning example and return to step 1 until all the m examples are trained;

7. reselect a set of input-output example randomly from m training samples and return to step (3) until the global error e of the network is smaller than the stopping threshold. If the learning epochs are greater than M, it means that the BP network fails to converge.

#### 3.2.2. Determination of Input and Output Factors

There are several criteria that should be taken into consideration when choosing input factors, such as the measurability, the importance, and relevance to the output factor, and data availability and credibility. Groundwater in the study area is recharged by rainfall and irrigation return flow, and is discharged mainly through artificial exploitation for agricultural, industrial, and domestic supply. Agriculture is the largest consumer of groundwater, accounting for over 75% of the total water consumption, followed by industrial consumption with an increasing proportional share of about 15%. Domestic supply accounts for the smallest proportion, approximately 10%. In official statistics, irrigation water consumption is determined by irrigation quota, which is estimated based on the amount of rainfall and its temporal distribution. However, the actual irrigation water consumption is closely related to the planting density, soil quality, crop yield, and irrigation methods [33]. Therefore, the statistics of irrigation water consumption may differ considerably from the actual value. For agriculture, previously, water-consuming crops including winter wheat, summer corn, peanuts, and fruit trees are widely planted in the study area. In order to conserve the limited groundwater resources, the local government has adapted the crop-planting structure to replace water-intensive crops with water-efficient verities. In addition, there are numerous small and medium-sized rural enterprises and family workshops in the study area, and many of them are not equipped with water meters. In view of the analysis above, the following input factors were selected: annual rainfall (×1), annual average temperature (×2), irrigated area (×3), water-saving irrigated area (×4), sown area of high-water-consumption crops (×5), gross industrial output value index (×6), and reservoir water supply (×7). Among these factors, the annual rainfall (×1) and the annual average temperature (×2) represented natural factors, and the other five factors were human-related factors. The output factor was the annual groundwater level change.

### 3.3. Sensitivity Analysis

In order to determine the dominance of the factors, the sensitivity analysis was performed based on default factor analysis and grey relational analysis (GRA) separately. By removing each input factor from the full-factor BP model individually, the sensitivity of the groundwater level to the removed factor can be calculated by Equation (4):

(4) $$R_{i}=MAE_{i}/MAE$$

Where, *R_i_* is the sensitivity index. If *R_i_* > 1, factor *i* is a sensitive factor. Otherwise, it is considered a redundant factor; *MAE* and *MAE_i_* are the mean absolute error of the full factor model and the default factor model after factor *i* is removed, respectively. If *MAE_i_ > MAE_j_*, it implies that the groundwater level is more sensitive to factor *i* than factor *j*.

The fundamental of GRA is to measure the correlation degree of data sequences in line with the similarities between the geometries of the data sequence curves [34]. For n data sequences with m influencing factors, create the comparison data sequences $\left\{x_{i}\right\}=\left\{x_{i}\left(1\right),x_{i}\left(2\right),\ldots ,x_{i}\left(n\right)\right\},i=1,2,\ldots ,m;$ and the reference data sequence {y}={y(1),y(2),…,y(n)}. To make comparison between values of different dimensions and orders of magnitude, the data sequences should be normalized using with Equation (5):

(5) $$x_{i}^{\prime }\left(k\right)=\frac{x_{i}\left(k\right)}{x_{i}\left(1\right)},\;y^{\prime }\left(k\right)=\frac{y\left(k\right)}{y\left(1\right)}$$

Calculate the absolute difference between the corresponding elements of the new comparison data sequences $\left\{x_{i}^{\prime }\right\}$ and the new reference data sequence $\left\{y^{\prime }\right\}$ with Equation (6):

(6) $$\Delta_{i}\left(k\right)=\left|x_{i}^{\prime }\left(k\right)-y^{\prime }\left(k\right)\right|$$

Find the maximum and minimum values from $\left\{\Delta_{i}\right\}$ using Equation (7)

(7) $$\Delta_{max}=\underset{i=1}{\max}\underset{k=1}{\max}\left|x_{i}^{\prime }\left(k\right)-y^{\prime }\left(k\right)\right|,\;\Delta_{min}=\underset{i=1}{\min}\underset{k=1}{\min}\left|x_{i}^{\prime }\left(k\right)-y^{\prime }\left(k\right)\right|$$

Calculate the grey relational coefficient of the corresponding elements of each influencing factor sequence and target variable sequence with Equation (8):

(8) $$r_{i}\left(k\right)=\frac{\Delta_{min}+\rho \Delta_{max}}{\left|x_{i}^{\prime }\left(k\right)-y^{\prime }\left(k\right)\right|+\rho \Delta_{max}}$$

Where, ρ is the resolution coefficient, ρ ∈ (0,1). Here, ρ takes 0.5.

The grey relational grade between impact factor i and the dependent variable can be obtained by calculating the average value of grey relational coefficients with Equation (9):

(9) $$R_{i}=\frac{\sum_{k=1}^{n}r_{i}\left(k\right)}{n}$$

R ∈ [0,1]. The closer of R to 1, the more sensitive the dependent variable to the influence factor is.

### 3.4. Hydrochemical Diagrams and ion Ratio Coefficients

This paper used the Piper diagram combined with the descriptive statistics to assess groundwater hydrochemistry in the research area. Further, the origin of groundwater, the sources of groundwater components, and the main hydrogeochemical processes controlling groundwater evolution were discussed with the ion ratio coefficient method and Gibbs diagram. Groundwater formed under different sources and conditions often exhibits specific ionic ratios. Therefore, some ion ratio coefficients can be used to predict the formation of groundwater and the origin of its components. Specifically, γNa^+^/γCl^-^ was used in this paper to determine if groundwater was marine origin connate water or derived from precipitation [35,36,37]; γ(Ca^2+^+Mg^2+^)/γ(${\mathrm{HCO}}_{3}^{-}$+${SO}_{4}^{2-}$) and γCa^2+^/γMg^2+^ was used to infer the sources of groundwater Ca^2+^ and Mg^2+^ in [38,39,40,41]; γ(Na^+^-Cl^−^)/γ[(Ca^2+^+Mg^2+^)-(${HCO}_{3}^{-}$+${SO}_{4}^{2-}$)] was used to indicate the existence of water-rock cation exchange reactions. Further, chloro-alkaline indices (CAI) was used to determine the direction of cation exchange [42,43], which is obtained by Equation (10):

(10) $$CAI-1=Cl^{-}-\left[\frac{\left(Na^{+}+K^{+}\right)}{Cl^{-}}\right]\;CAI-2=Cl^{-}-\left[\frac{\left(Na^{+}+K^{+}\right)}{SO_{4}^{2-}}\right]+HCO_{3}^{-}+CO_{3}^{2-}+NO_{3}^{-}$$

Negative values of both CAI-1 and CAI-2 imply that Ca^2+^ and Mg^2+^ in groundwater exchange with Na^+^ and K^+^ in the rocks, while positive values suggest the reversed cation exchange reaction.

### 3.5. Fuzzy Clustering

Cluster analysis is a useful statistic tool for grouping data. There are two categories of clustering algorithms, namely hard clustering and fuzzy clustering. For hard clustering, each sample should belong to exactly one cluster. While for fuzzy clustering, the samples can potentially be assigned to multiple clusters with membership degrees in the interval [0,1].

Most real-world classes of objects are fuzzy in nature. Generally, the physicochemical properties of groundwater gradually change as it moves along its flow path. As the category boundaries are almost never clear-cut, fuzzy clustering is highly suitable [24]. Fuzzy C-means clustering (FCM) has been adopted in many cases for river, lake, and ocean water quality evaluation [44,45,46,47]. However, there are few applications in the field of groundwater quality classification. In this work, FCM clustering is used to identify the spatial variation of groundwater environment in the study area to reveal the driving role of human activities and natural processes on coastal groundwater ecological environment evolution.

Herein introduce the basic principle of FCM. For data set ${\left\{X_{j}\right\}}_{j=1}^{n}$, the algorithm aims to group ${\left\{X_{j}\right\}}_{j=1}^{n}$ into c clusters, and find the clustering center of each cluster to minimize the objective function describing the dissimilarity of the data in the same cluster. The objective function is expressed as Equation (11):

(11) $$F_{m}\left(U,C\right)=\overset{c}{\underset{i=1}{\sum }}\overset{n}{\underset{j=1}{\sum }}{\left(u_{ij}\right)}^{m}{\left(d_{ij}\right)}^{2}$$

Where, U and C are the membership matrix and the cluster center matrix, respectively; c is the number of clusters; u_ij_ is the membership degree of x_j_ to cluster center c_i_, $\sum_{i=1}^{c}u_{\mathrm{ij}}=1(u_{\mathrm{ij}}\in \left[0,\;1\right],\;\forall j=1,\;2,\;\ldots ,\;n,\;0<\sum_{j=1}^{n}u_{\mathrm{ij}}<n)$; m is the weight exponent, m = [1, +∞). As m controls the degree of data sharing, it largely determines level of fuzzy overlap between clusters; d_ij_ is the distance from x_j_ to the category center c_i_. The Euclidean distance was used in this paper.

The problem is translated into finding an optimal solution of the objective function under the constrain of $\sum_{i=1}^{c}u_{ij}=1$. With Lagrange multiplier method, c_i_ and u_ij_ can be obtained as Equation (12):

(12) $$c_{i}=\frac{\sum_{j=1}^{n}{\left(u_{ij}\right)}^{m}x_{j}}{\sum_{j=1}^{n}{\left(u_{ij}\right)}^{m}}\;u_{ij}={\left\{\overset{c}{\underset{k=1}{\sum }}{\left(\frac{d_{ij}}{d_{kj}}\right)}^{\frac{2}{m-1}}\right\}}^{-1}$$

Iterative optimization is applied to find the optimum C and U to minimize F_m_(U, C). The steps are as follows:

1. predefine the cluster number c and weight exponent m. Initialize the fuzzy membership matrix U under the constrain of $\sum_{i=1}^{c}u_{ij}=1$;

2. calculate the cluster centers with Equation (10);

3. use Equation (11) to calculate the memberships of samples to the clusters to update the fuzzy membership matrix U;

4. if the difference of F_m_(U, C) between two successive iteration is smaller than the given threshold, stop the loop. Otherwise, go back step 2.

The values of fuzzy cluster c and weight exponent m can greatly affect the performance of FCM. A derivative function −[(δF_m_(U, C)/δm)*c*^0.5^] against m is devised to find the optimal parameter combination of c-m [48]. For different c against a series of m, the one that minimize the maximum value of the function is the most valid. When the value of c is determined, the m at the peak of the function is the optimal choice. −[(δF_m_(U, C)/δm)*c*^0.5^] can be obtained by Equation (13):

(13) $$\frac{\delta \mathrm{Fm}\left(U,\;C\right)}{\delta m}=\overset{n}{\underset{j=1}{\sum }}\overset{c}{\underset{i=1}{\sum }}[\left(u_{ij}{)}^{m}\times {\left(d_{ij}\right)}^{2}\times \mathrm{lg}\left(u_{ij}\right)\right]$$

Further, the fuzzy performance index (FPI) and normalized classification entropy (NCE) were introduced to identify the optimal cluster number and assess the effectiveness of the classification result [46,49]. The best number of clusters is determined when FPI and NCE are both at a minimum. FPI measures the separation between clusters which can be calculated by Equation (14):

(14) $$FPI=1-\frac{c\times \frac{\sum_{j=1}^{n}\sum_{i=1}^{c}{\left(u_{ij}\right)}^{2}}{n}-1}{c-1}.$$

FPI ∈ (0, 1). A value tending to 0 suggests that there is less shared data and greater difference between clusters, which means the classification is more clear and valid. Whereas a value tending to 1 suggests more significant overlap between the clusters. Generally, for a given m, the local minimized FPI along a series of c points to the best clustering for this m [47].

NCE is used to estimate the disorganization degree created by a given number of clusters, which is obtained by Equation (15):

(15) $$\mathrm{NCE}=\frac{\left[-\frac{\sum_{j=1}^{n}\sum_{i=1}^{c}u_{ij}lg\left(u_{ij}\right)}{n}\right]}{1-c/n}.$$

NCE ∈ (0, 1). A greater value of NCE indicates higher level of disorganization of the classification [50].

In view of unclear boundaries of clusters, the confusion index (CI) was introduced to evaluate the uncertainty of cluster membership [51], which is calculated by Equation (16):

(16) $$\mathrm{CI}=1-\left[u_{maxj}-u_{\left(max-1\right)j}\right]$$

Where, u_maxj_ and u_(max − 1)j_ are maximum membership value and the second largest membership value of x_j_ for the clusters, respectively.

## 4. Results

### 4.1. BP-ANN Model for Groundwater Level Dynamics and Factor Sensitivity Analysis

**Figure 6:**
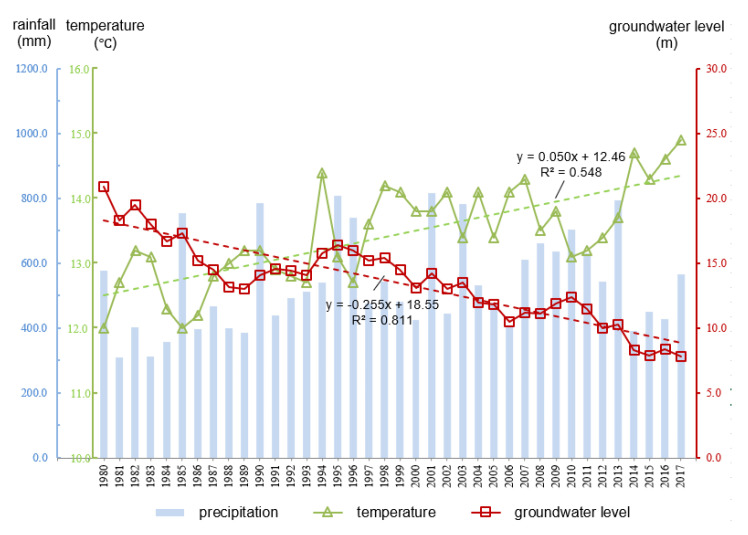
Annual rainfall, average annual temperature, and average annual groundwater level variation in the study area from 1980 to 2017.

Figure 6 displays the changes of annual rainfall, average annual temperature and average annual groundwater level in the study area from 1980 to 2017 (data were supported by Yantai Hydrological Bureau. The location of the meteorological station is shown in Figure 1, and the location of groundwater level monitoring wells is shown in Figure 5. Rainfall varied widely from year to year, and consecutive dry years occurred frequently. The temperature followed an upward trend, at the climate trend rate of 0.499 °C/10a (*p* < 0.01). The average annual groundwater level dropped 13.16 m with a descent rate of 0.379 m/a. During the 1980s, due to the consecutive years of drought and the substantial increase in groundwater consumption, groundwater level declined dramatically, with an average drop of 6.82 m over 10 years. After 1990, although groundwater levels are on an overall downward trend, the rate of decline has slowed. This should be attributed to the upturn of rainfall and the implementation of water saving and groundwater artificial recharge techniques [52,53,54].

**Figure 7:**
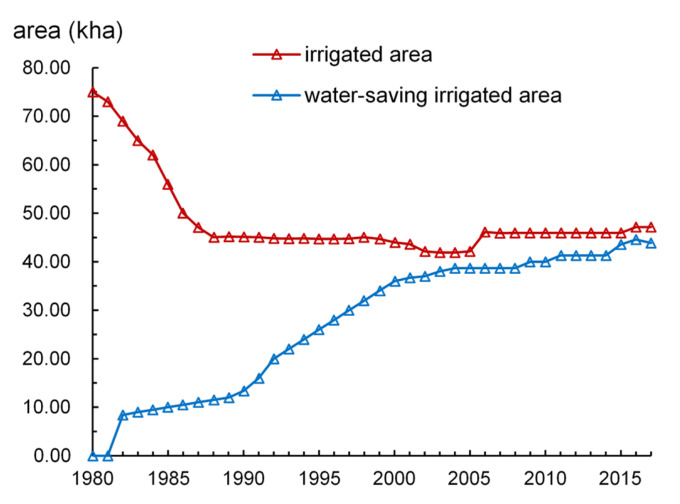
Irrigated area and water-saving irrigated area in the study area from 1980 to 2017.

**Figure 8:**
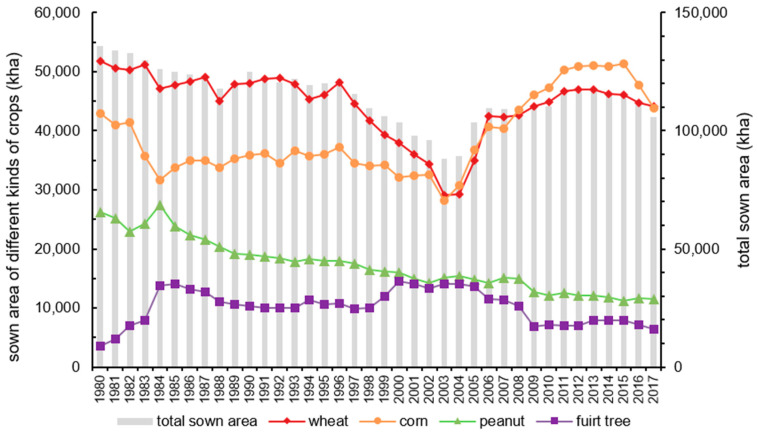
Sown area of main crops in the study area.

**Figure 9:**
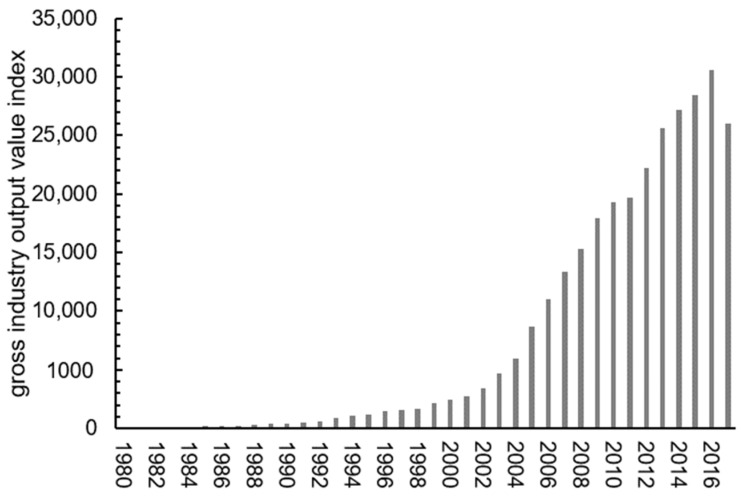
Gross industrial output value index (1980 Index = 100).

**Figure 10:**
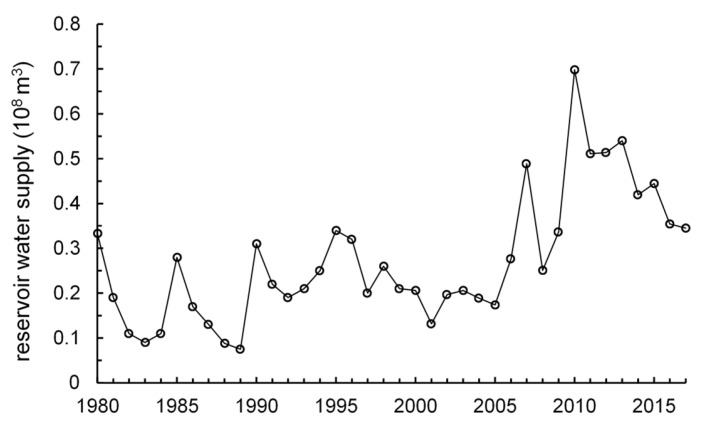
Reservoir water supply.

The irrigated area and water-saving irrigated area in study area from 1980 to 2017 is presented in Figure 7. From 1980 to 1990, due to the shrink of cultivated land area and reduced rainfall, the effective irrigation area in the study area decreased continuously [55], and became stable after 2005. With the implementation of water-saving technology, the area of water-saving irrigation has been gradually increasing. Figure 8, Figure 9 and Figure 10 (data supported by Yantai Statistics Bureau) show the sown area and of crops, the gross industrial output value index and reservoir water supply in the study area. The sown area of water-intensive crops decreased but rose again. The gross industrial output value index experienced a rapid growth. Reservoir water supply fluctuated greatly, but had an upward trend.

### 4.2. Preprocessing of Training Samples

The magnitude difference between the input factors will lead to decreased importance of lower magnitude factors to the system output, and affect the learning speed and accuracy of the model. Therefore, the standard raw method was used to process the actual original input data to [0,1] interval [32].

### 4.3. Model Construction and Training

**Figure 11:**
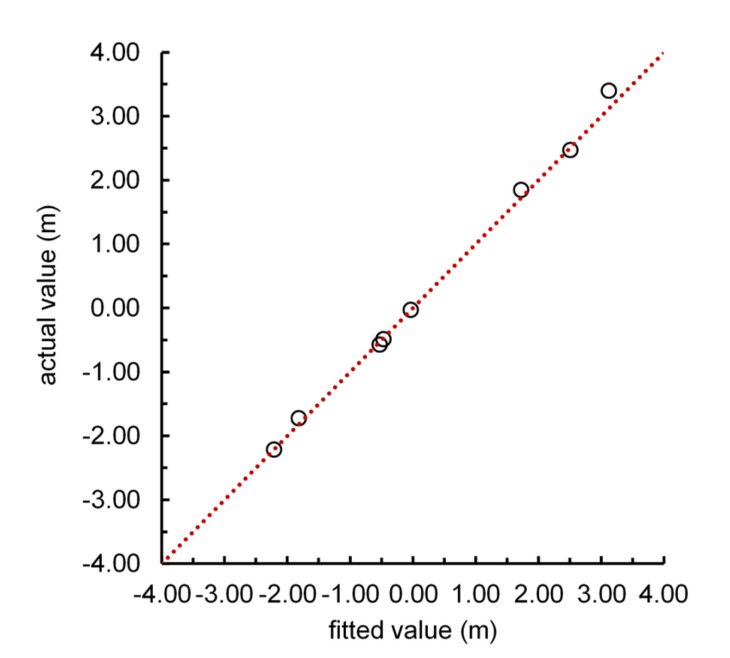
Comparison between the fitted and measured values of the annual change of groundwater level.

The hyperbolic tangent transfer function and linear transfer function were used respectively in the hidden layer and output layer. Trainlm was selected as the training function. Moreover, 38 sets of data from year 1980 to 2017 were randomized into 30 training data sets and 8 test data sets. The number of hidden layer neurons was identified to be 8 after the model went through repeated adjustments. The error convergence performance was good during training, and the model fitted the data well. The 8 test datasets were used to prove the accuracy of the established model. The results (Figure 11) showed that the absolute value of the model average error was 0.069 m, the maximum relative error was 9.11%, and the absolute value of the average relative error was 3.82%. It can be seen that although there was less training data for modeling, the model achieved a satisfactory degree of accuracy. The disadvantage of the method lies in that due to the limitation of data availability, the selected factors may not be reasonable, and the established model is not conducive to the analysis of the internal relations between the factors. It is impossible to analyze the relationship between groundwater dynamics and the factors at the mechanism level.

### 4.4. Sensitivity Analysis

The sensitivity analysis was performed with default factor test method and GRA separately for cross-validation. In order to make the default factor model comparable to the full factor model, the learning algorithm of the network, as well as the training and test samples used in the default factor model were the same as that in the full factor model. As can be seen from Table 1, in comparison with the full factor model, the test errors of the default factor models were greater, which meant the groundwater level was sensitive to all of the input factors. By calculating the sensitivity ranking, the sensitivity of the groundwater level to the input factors were obtained: irrigated area > water saving irrigated area > annual precipitation > gross industrial output value index > sown area of high-water-consumption crops > average temperature > reservoir water supply. 

The results of GRA method (Table 2) showed were slightly different from that of default factor test method (Table 1). The impact factors were in the order of irrigated area > water saving irrigated area > annual precipitation > gross industrial output value index > sown area of high-water-consumption crops > reservoir water supply > average temperature. This suggested that human activities were the more important driving factors for the dynamic changes of groundwater levels in the study area. Agricultural activities have the greatest impact on the groundwater level. Water saving irrigation and the promotion of less water-consumption crops are effective ways to save groundwater.

### 4.5. Groundwater Hydrochemical Characteristics

**Figure 12:**
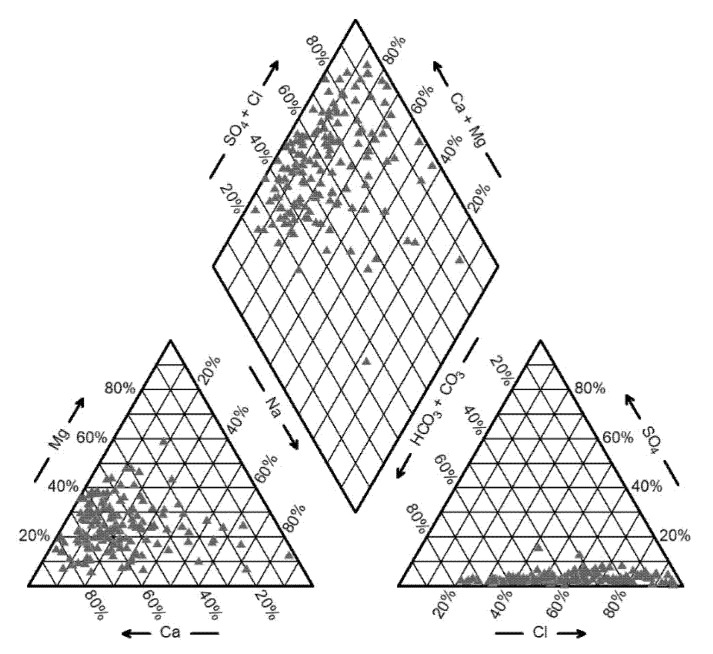
Piper diagram of groundwater samples.

Figure 12 and Table 3 shows the hydrochemical types and statistic values of major groundwater ions of the groundwater samples. The groundwater hydrochemical type in low mountainous hilly areas was mainly magnesium bicarbonate type. The dominant cation and anion in groundwater were Ca^2+^ and ${HCO}_{3}^{-}$, and the TDS was relatively low. Groundwater was less vulnerable to contamination due to the small population density and underdeveloped industry and agriculture. While in the plain area, due to the strong evaporation, intense human activities and seawater intrusion, the groundwater TDS increased from inland towards the sea, and the dominant ions in groundwater gradually changed from Ca^2+^ and ${HCO}_{3}^{-}$ to Na^+^ and Cl^−^. The coefficients of variation of Na^+^+K^+^, Ca^2+^, Mg^2+^, Cl^−^ and ${SO}_{4}^{2-}$ were greater than 1. This indicated that the contents of these ions varied greatly over the area, which were largely dependent on hydrogeological conditions, topography, hydrometeorology, and human activities. Table 4 shows that Cl^−^ showed a strong positive correlation with Na^+^+K^+^, Ca^2+^, and Mg^2+^. Mg^2+^ had a strong correlation with ${SO}_{4}^{2-}$. TDS has a strong correlation with Na^+^+K^+^, Ca^2+^, Mg^2+^, Cl^−^ and ${SO}_{4}^{2-}$.

**Figure 13:**
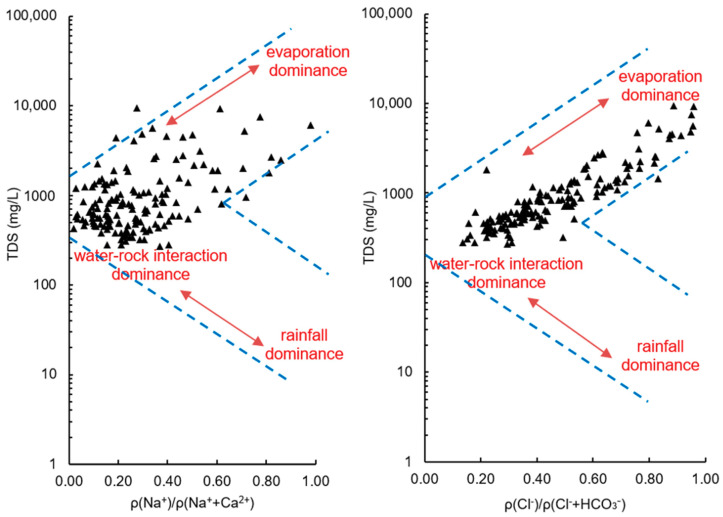
Gibbs diagram of the groundwater samples.

Based on the Gibbs diagram, ion ratios and hydrogeological conditions, the sources of some major ions in groundwater and the main hydrogeological processes in control were discussed. Under natural conditions, the chemical composition of groundwater is the product of precipitation, water-rock interaction, and transpiration. Gibbs diagram is a powerful tool to identify the major processes governing groundwater chemistry [56]. Water samples located on the bottom right corner of the plot have higher ρ(Na^+^)/ρ(Na^+^+Ca^2+^) or ρ(Cl^−^)/ρ(Cl^−^+${HCO}_{3}^{-}$) ratio and lower TDS level, and the compositions are likely to be controlled by precipitation. Those on the left central zone of the plot are characterized by low ρ(Na^+^)/ρ(Na^+^+Ca^2+^) or ρ(Cl^−^)/ρ(Cl^−^+${HCO}_{3}^{-}$) ratio and moderate level of TDS, indicating the dominance of rock weathering. Correspondingly, under intensive evaporation conditions, the samples with high TDS and high ratio of ρ(Na^+^)/ρ(Na^+^+Ca^2+^) or ρ(Cl^−^)/ρ(Cl^−^+${HCO}_{3}^{-}$) fall within the upper right part of the plot. The Gibbs diagram of the groundwater samples in the study area (Figure 13) clearly shows that all the samples were away from the precipitation dominance zone. This strongly suggested that atmospheric precipitation was not the predominant controlling factor for groundwater hydrochemistry over the study area. Groundwater samples collected from hilly areas, which had low TDS, were located in the rock weathering dominance zone. While for samples in the coastal plain area, ρ(Na^+^)/ρ(Na^+^+Ca^2+^) and ρ(Cl^−^)/ρ(Cl^−^+${HCO}_{3}^{-}$) ratios and TDS were much higher, indicating that evaporation-crystallization was the key process. This was consistent with the characteristics of intense evaporation and seawater intrusion in the coastal region.

**Figure 14:**
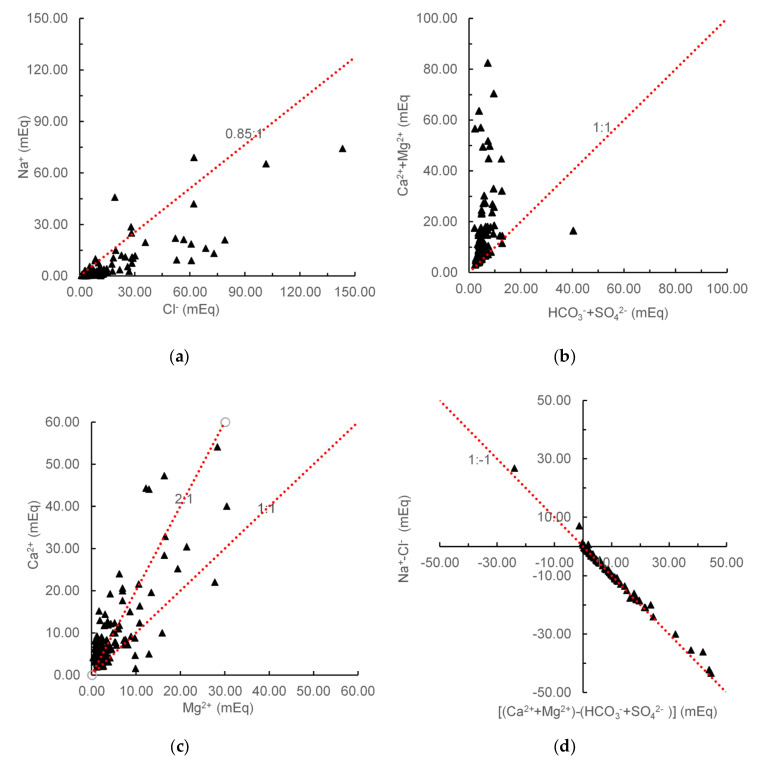
Ion ratios of groundwater samples. (**a**) γNa^+^/Cl^−^, (**b**) γ(Ca^2+^+Mg^2+^)/γ(${HCO}_{3}^{-}$+${SO}_{4}^{2-}$), (**c**) γCa^2+^/γMg^2+^, (**d**) γ(Na^+^-Cl^−^)/γ[(Ca^2+^+Mg^2+^)-(${HCO}_{3}^{-}$+${SO}_{4}^{2-}$)].

As shown in Figure 14a, γNa/γCl ratios of groundwater samples were generally less than 0.85, indicating that there was widespread cation exchange between Na^+^ in groundwater and exchangeable Ca^2+^ in the rocks. The values of γ(Ca^2+^+Mg^2+^)/γ(${HCO}_{3}^{-}$+${SO}_{4}^{2-}$) were generally greater than 1, which demonstrated that Ca^2+^ and Mg^2+^ in groundwater were mainly from carbonate, sulfate and silicate (Figure 14b). The γCa^2+^/γMg^2+^ ratios further illustrated that the main carbonate minerals included dolomite, calcite, and silicates (Figure 14c). As depicted in Figure 14d, γ(Na^+^-Cl^−^) and γ[(Ca^2+^+Mg^2+^) − (${HCO}_{3}^{-}$+${SO}_{4}^{2-}$)] showed a strong negative correlation with a slope of about −1, which meant that cation exchange reactions played an important role in the formation of groundwater components. 

**Figure 15:**
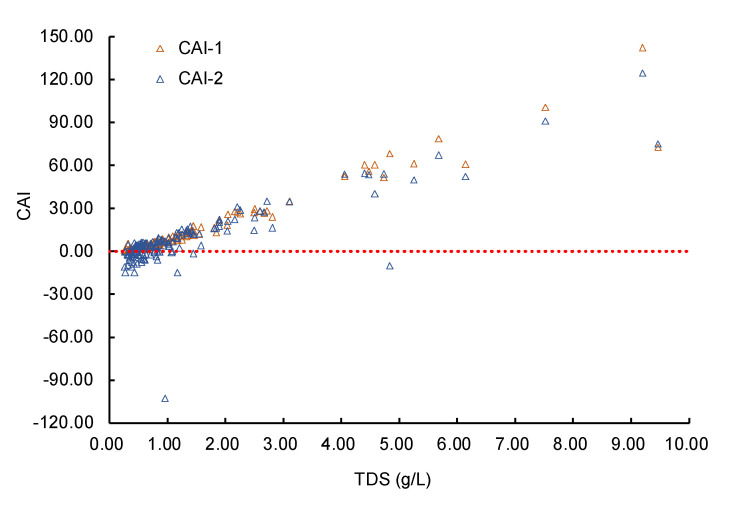
The chloro-alkaline indices of groundwater samples.

Figure 15 describes the chloro-alkaline indices of the groundwater samples. The CAI-1 values of all the sampling were positive. For CAI-2, when TDS < 1 g/L, the values of most sampling points were negative. As TDS increased, both CAI-1 and CAI-2 had an upward trend, and CAI-2 were generally greater than 0. This provides compelling evidence that the reverse cation exchange reaction between the ions in groundwater (Na^+^ and K^+^) and the ions in rock (Mg^2+^ and Ca^2+^) was an important reason for the increase of TDS.

### 4.6. Groundwater Quality Classification Based on Fuzzy Clustering

**Figure 16:**
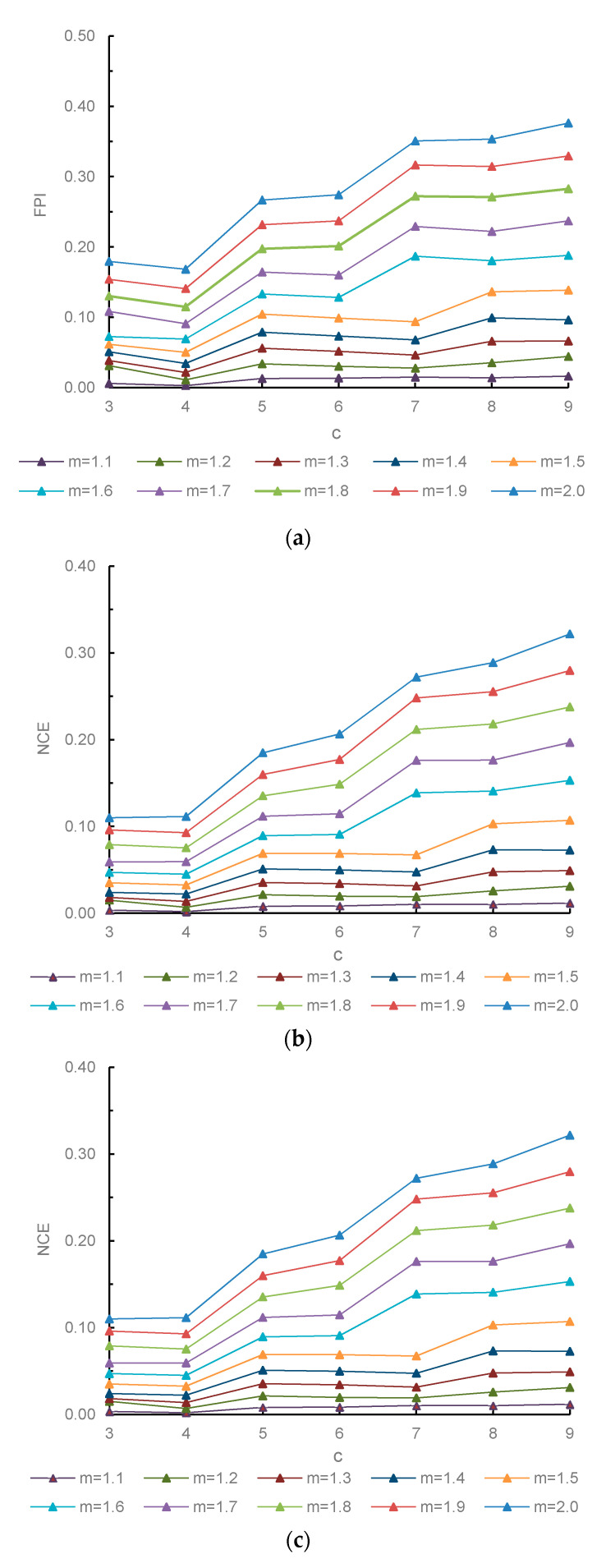
FPI, NCE, and derived function curves with different combinations of c-m (c is the cluster number, m is the weighting exponent). (**a**) Variation of fuzzy performance index (FPI) along m with different c. (**b**) Variation of normalized classification entropy (NCE) along m with different c. (**c**) Variation of derived function along c with different m.

**Figure 17:**
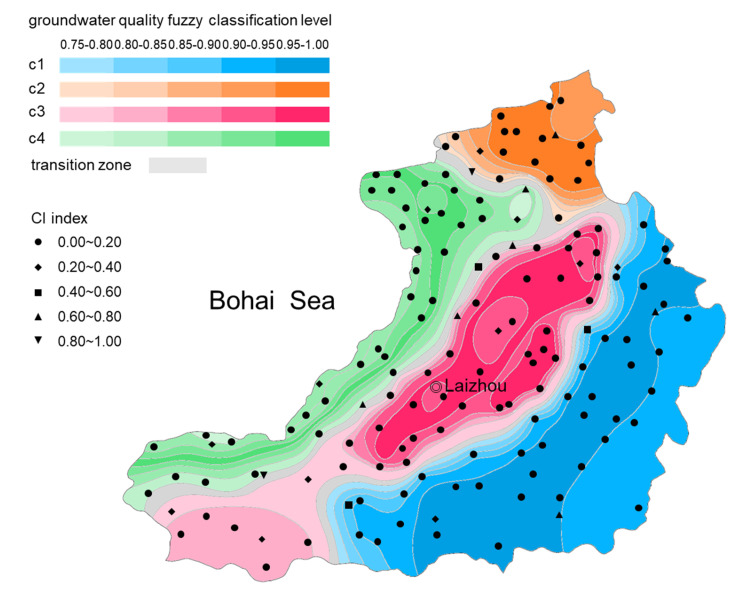
Confusion index and groundwater quality fuzzy classification results.

As shown in Figure 16, the minimum peak value of the curve was reached at c = 4 with m = 1.9. The FPI and NCE functions also reached their minimum value at c = 4. Therefore, the classification number c and fuzzy exponent m were determined to be 4 and 1.9, respectively. Figure 17 shows that each partition was spatially continuous. The confusion index of most sampling was less than 0.2, and the average confusion index of the study area was 0.119. This indicated that the overlapping degree of different fuzzy categories was small, and the subordination relationship of groundwater types was relatively clear. If the maximum degree of membership of the sample is greater than 0.75, the class with the highest degree of membership of the sample was regarded as the classification to which it belonged. Otherwise, the sample fell into the transitional category.

Table 5 presents the cluster centers and the average concentrations of the groundwater quality indicators in each cluster. It can be seen that there was great difference in groundwater chemistry characteristics between the clusters. The groundwater samples of c1 cluster were mainly located in the eastern and southeastern hilly areas. The samples of this cluster had low TDS and pollutant concentration levels. In this zone, the groundwater environment was less affected by human activities due to the small population density and underdeveloped agriculture and industry. Among the 47 samples, 41 of them met the Grade III Water Quality Standard of National Standards of People’s Republic of China—Groundwater Quality Standards (GBT 14848-2017). However, the content of Zn, COD_Mn_ and NH+ 4 of 3 samples, Cr^6+^, Pb, Zn, cyanide and volatile phenol of 1 sample, as well as Zn and NH+ 4 of 2 samples exceeded the Grade III water quality standard. These sampling points were located nearby the poultry farms and tailings ponds. As the feed additives are extensively used in intensive animal farms, the poultry manure usually contains high levels of heavy metals such as Zn. The groundwater can be contaminated by the untreated wastewater from the poultry farms. The tailings ponds store the tailings slag of the gold mine, which contains substantial toxic substances including heavy metals, cyanides, and volatile phenols. The seepage from the tailing ponds is a potential contaminant of groundwater.

The groundwater samples of cluster c2 concentrated in the northern part of the study area. These samples generally had low pH values and high concentrations of Cr^6+^, Pb, Zn, cyanide, and volatile phenol. Of the 16 samples, only 5 of them met the Grade III water quality standard. There were 4 samples with pH values less than 6.5, 9 samples with Cr^6+^ concentration exceeding 0.05 mg/L, 7 samples with Pb content exceeding 0.01 mg/L, 11 samples with Zn content exceeding 1.00 mg/L, 8 samples with cyanide content exceeding 0.05 mg/L, and 8 samples with volatile phenols content over 0.002 mg/L, which were inferior to the Grade III water quality standard. This area is rich in gold and iron ore resources, and most of them are associated with pyrite and other sulfides. Groundwater contamination in this region was mainly attributed to mining wastewater, which contains a significant amount of organic pollutants, oil, cyanide, acids, heavy metals, fluorides, and soluble salts. 

Groundwater samples belonging to cluster c3 were mainly located in the central plain of the study area, where the population density is large and the industry and agriculture are well developed. Groundwater in this region was characterized with extremely high TDS, and the ${NO}_{3}^{-}$ content, total coliforms and COD_Mn_ were much higher than those in other parts of the study area. Some sampling points were located near small township factories, such as printing and dyeing factories, food processing plants, paper mills, brick and tile factories, and rural landfills. The concentrations of Cr^6+^, Zn, ${NH}_{4}^{+}$, cyanide, volatile phenol, and COD_Mn_ concentrations of these samples exceed Grade III water quality standard. This indicated that the shallow groundwater in this zone was affected by agricultural irrigation (nitrogen fertilizer application, etc.), domestic and industrial discharge. First, some villages are not equipped with garbage collection, transportation, landfill, and treatment system. The solid waste collected and deposited in open dumps or landfills are subjected to infiltration from precipitation, or any other possibility of infiltration of water, which has led to groundwater contamination. Second, the central plain is the important agricultural production zone. The main cropping system is winter wheat-summer maize rotation, which is characterized by high fertilizer input. The field investigations found that the average application rate of nitrogen fertilizer per hectare for this cropping system is around 850 kgN/ha. However, the utilization rate of nitrogen fertilizer is only 10–20%, and approximate 25% of the total applied N is leached into the aquifer. Furthermore, the wastewater from the numerous rural enterprises is discharged into river and ground with insufficiently treatment. Table 6 shows the pollutant content of wastewater samples collected from sewage outlets of four factories:

The samples of cluster c4 are concentrated in the coastal plain. Due to seawater intrusion, groundwater Cl^−^, Na^+^+K^+^ content and TDS level were far higher than the other zones. The average Cl^−^ and Na^+^+K^+^ concentrations were 1166.16 mg/L and 362.60 mg/L, and the average TDS reached 2.45 g/L. In addition, high levels of ${NO}_{2}^{-}$and ${NH}_{4}^{+}$ were noticed in the offshore area, which was presumed to be caused by the aquaculture wastewater. Intensive aquaculture, characterized by high stocking density, high input of feed, fertilizer and fishery medicine, has developed enormously in the offshore area since 1990s. The aquaculture wastewater usually contains high concentrations of ${NH}_{4}^{+}$-N, which is derived from residual baits, fishery fertilizer, animal metabolites, and biological debris. Generally, the effluent of the farms is discharged directly into open trenches or the sea or without recirculation or any further treatment. In addition, the substantial use of disinfectants basis and oxygen deficiency leads to the hindered nitrification and the ammonia and nitrite enrichment. As most of the earthen ponds and drainage ditches were not impervious, the wastewater rich in ${NO}_{2}^{-}$-N and ${NH}_{4}^{+}$-N easily penetrated into the groundwater. Furthermore, pollutants from aquaculture can migrate upstream with salt water. The concentrations of N pollutants of samples collected from aquaculture ponds, drainage ditches and offshore waters were listed in Table 7. It has been proved that the nitrogen derived from mariculture fertilizer mainly accounts for the ${NH}_{4}^{+}$-N contamination in the central plain area [57]. 

## 5. Discussion

The coastal groundwater environment is fragile and sensitive to changing environment. With shifting climate pattern and enhancement of human activities, coastal areas are exposed to threats of groundwater environmental issues, such as decreasing groundwater levels, seawater intrusion, and groundwater pollution. Understanding the magnitude of impact of natural and human factors on groundwater environment, as well as the process and mechanism of groundwater environment evolution can provide technical supports for coastal groundwater environmental protection and rehabilitation. Previous researches have focused on the response of changing environment on groundwater quantity or quality. This study integrated the impacts of changing environment on both groundwater level dynamics and groundwater quality.

In areas where human activities are intensive, the impact of human activities on groundwater environment has been increasing to an even greater extent than that of natural factors, is a conclusion shared by many researchers. One of the most important finding of this research is that human factors including irrigated area and water-saving irrigated area were the most important influencing factors on groundwater level dynamics, followed by annual precipitation. This is reasonable in areas where agricultural water consumption accounts for the most significant proportion of groundwater extraction. Zhang, Z.Z. et al. [58] analyzed the impacts of precipitation, evaporation, agricultural and industrial exploitation, and domestic water consumption to groundwater depth in an agriculture-dominated area in Northeast, and the results discovered that agricultural exploitation ranked first among all influencing factors. Liu, J. et al. [18] also found that human activities had the primary role in groundwater dynamics in an irrigated region in Xinjiang, China, among which the increase of water- saving irrigated area with groundwater as the main water source was the main reason for groundwater level decline.

The results of groundwater origin and main hydrogeological process in control in the study area were consistent with the meteorological, geological landform, and hydrogeological conditions of the study area. The groundwater samples were classified into four clusters based on their hydrogeochemical signatures with the fuzzy C-means method. Each cluster was spatially continuous, and the spatial variation of groundwater quality could be well interpreted by the types and intensity of human activities. However, this study is cursory in its analysis of the causes of groundwater quality differences and pollution. Statistical methods need to be used to extract the main factors responsible for water quality variation. For example, Güler, C. et al. [24] employed Principal Components Analysis to interpret the underlying natural & anthropogenic factors contributing to groundwater quality differences.

Considered a black box hydrological model by most users, the BP model can be used effectively for finding the causal relationship between the influencing factors and groundwater level dynamics. The model cares about the accuracy of the simulation results. It is impossible to analyze the relationship between groundwater dynamics and the factors at the mechanism level, or to describe the detailed hydrological process. While applying the BP model for prediction, the results may not be very accurate if the data is close to or exceed the boundary of specific data used for training. Another disadvantage of the method lies in that due to the limitation of data availability, the selected factors may not be reasonable. In this paper, the data used for training were selected randomly from the data set. The established BP model achieved a satisfactory degree of accuracy (as demonstrated in Figure 11).

The other shortcoming of this work is that the seasonal variation of groundwater components cannot be analyzed as only one sampling was conducted. However, previous studies in the research area have shown that groundwater pollutants may vary significantly in different seasons due to precipitation, agricultural activities including fertilization and irrigation, and denitrification [53,55]. In addition, the main hydrogeochemical processes that influence the groundwater chemistry may also change with seasons. Kumar, M. et al. [59] noticed the periodic seasonal switchover in the hydrogeochemical processes in Delhi, India. The study in Bou-Areg Aquifer, Morocco, also verified that processes governing the aquifer’s hydrochemistry varied in irrigation and non-irrigation periods as a result of natural factors and human activities such as irrigation [5]. Thus, continuous monitoring of groundwater quality should be strengthened to highlight seasonal variations when evaluating groundwater quality.

In addition, as the rivers dried up during the sampling period, no river water samples were taken, which is not conductive to the evaluation of the sources of groundwater pollution. In addition, the natural background values for groundwater should be analyzed. In the evaluation of groundwater pollution, it is necessary to exclude the deterioration of groundwater quality caused by natural factors to make the evaluation of the impact of human activities on groundwater quality more accurate.

## 6. Conclusions

This article takes the eastern coast of Laizhou Bay as an example to analyze the response of groundwater environment to changing environment. The main research contents and conclusions are as follows:

(1) the groundwater level dynamics in the study area from 1980 to 2017, and the fluctuation of groundwater levels from typical groundwater observation wells were analyzed. The main external environmental factors that affect the dynamics of the groundwater level were extracted, including natural factors such as rainfall, temperature, and human factors such as irrigated area and water-saving irrigation area. BP-ANN was employed to model the response of groundwater level to the above environmental factors. The results show that despite the limited length of the data series, the model established still had a satisfactory accuracy. Further, the default factor test method and GRA were used to determine the degree of influence of the driving factors on groundwater level dynamics. The results indicated that human activities were the more significant driving factors for groundwater level change in the study area;

(2) a total of 153 ground water samples were collected from April to May, 2017. The physical and chemical characteristics and determine the main geochemical processes controlling groundwater formation and evolution analyze to have a better understanding of the natural background of groundwater environment. The main groundwater hydrochemical type in the hilly areas was magnesium bicarbonate type, and the dominant cation and anion were respectively Ca^2+^ and ${HCO}_{3}^{-}$. While in the plain area the dominant ions in groundwater changed from Ca^2+^ and ${HCO}_{3}^{-}$ to Na^+^ and Cl^−^. Gibbs diagram suggested that the groundwater in hilly areas was mainly controlled by water-rock interaction, while that in coastal plain area was by evaporation-crystallization. The ion ratios indicated that cation exchange played an important role in the formation of groundwater compositions; 

(3) based on understanding the natural background of the groundwater environment, FCM clustering was adopted to classify the groundwater samples based on their hydrochemical characteristics to reveal the spatial variation of groundwater quality in the research area. The classification number c and fuzzy exponent m were determined to be 4 and 1.9. The partitions were spatially continuous. The CI values of most samples are less than 0.2, and the average CI over the study area is 0.119, indicating that the classification is valid and clear. The groundwater chemical and pollution characteristics of different clusters varies considerably. The c1 cluster samples were mainly located in the eastern and southeastern hilly areas of the study area. Groundwater was characterized by low TDS and low pollutant content levels due to the rapid groundwater renewal rate, low population density and less developed agriculture and industry. Samples belonging to c2 cluster were concentrated in the northern part of the study area. The groundwater had low pH values, and the contents of Cr^6+^, Pb, Zn, cyanide, and volatile phenol were much higher than other regions. Mining wastewater should be responsible for the high content of groundwater acidification and heavy metals, cyanide and phenolic pollutants. Groundwater samples of c3 cluster were mainly located in the central plain area. Groundwater ${NO}_{3}^{-}$ content, total coliforms and COD_Mn_ were much higher, which strongly suggests shallow groundwater in this zone was affected by agricultural irrigation (nitrogen fertilizer application, etc.), domestic and industrial discharge. The c4 cluster groundwater samples are concentrated in the coastal plains. Due to seawater intrusion, groundwater Cl^−^, Na^+^+K^+^ content and TDS far exceeded those in other areas. In addition, high ${NO}_{2}^{-}$ and ${NH}_{4}^{+}$ levels were observed in the aquaculture area.

## Figures and Tables

**Table 1:** Sensitivity of groundwater level to the influencing factors with default test method.

Influencing Factors	Full Factor Model	Annual Precipitation (×1)	Average Temperature (×2)	Irrigated Area (×3)	Water-Saving Irrigated Area (×4)	Sown Area of High-Water-Consumption Crops (×5)	Gross Industrial Output Value Index (×6)	Reservoir Water Supply (×7)
mean absolute deviation	0.069	0.414	0.113	0.532	0.438	0.187	0.261	0.093
mean absolute value of relative error	3.82	16.58	7.15	26.45	20.66	10.32	14.22	5.22
R	/	4.34	1.87	6.92	5.41	2.70	3.72	1.37
sensitivity ranking	/	3	6	1	2	5	4	7

**Table 2:** Sensitivity of groundwater level to the influencing factors with GRA method.

Influencing Factors	Annual Precipitation (×1)	Average Temperature (×2)	Irrigated Area (×3)	Water-Saving Irrigated Area (×4)	Sown Area of High-Water-Consumption Crops (×5)	Gross Industrial Output Value Index (×6)	Reservoir Water Supply (×7)
grey rational grade	0.802	0.683	0.903	0.847	0.732	0.766	0.701
sensitivity ranking	3	7	1	2	5	4	6

**Table 3:** Statistic values of major groundwater ions, TDS, and pH (number of sampling points: 153).

Statistic Values	Na^+^+K^+^	Ca^2+^	Mg^2+^	Cl^−^	${SO}_{4}^{2-}$	${HCO}_{3}^{-}$+${CO}_{3}^{2-}$	TDS	pH
	(mg/L)	(mg/L)	(mg/L)	(mg/L)	(mg/L)	(mg/L)	(g/L)	
minimum value	1.57	32.06	3.89	32.08	2.94	123.83	8.61	6.13
maximum value	1708.79	1082.16	364.8	5078.16	237.6	2395.47	229.93	8.16
average	124.59	175.89	52	455.42	30.09	281.07	36.6	7.73
coefficient of variation	2.12	1.01	1.22	1.58	1.39	0.7	1.2	0.03

**Table 4:** Correlation coefficients between major groundwater ions and TDS (number of sampling points: 153).

Correlation Coefficients	Na^+^+K^+^	Ca^2+^	Mg^2+^	Cl^−^	${SO}_{4}^{2-}$	${HCO}_{3}^{-}$+${CO}_{3}^{2-}$	TDS
Na^+^+K^+^	1.000	0.515	0.693	0.829	0.747	0.425	0.801
Ca^2+^		1.0000	0.791	0.876	0.590	0.065	0.848
Mg^2+^			1.000	0.910	0.860	0.144	0.928
Cl^−^				1.000	0.790	0.122	0.969
${SO}_{4}^{2-}$					1.000	0.256	0.843
${HCO}_{3}^{-}$+${CO}_{3}^{2-}$						1.000	0.165
TDS							1.000

**Table 5:** Cluster centers and average concentrations of the groundwater quality indicators in each cluster.

Cluster Center	Number of Samples	Characteristic Values	Na^+^+K^+^	Ca^2+^	Mg^2+^	Cl^−^	${SO}_{4}^{2-}$	${\mathrm{HCO}}_{3}^{-}$+${\mathrm{CO}}_{3}^{2-}$	TDS	pH	COD_Mn_
			(mg/L)	(mg/L)	(mg/L)	(mg/L)	(mg/L)	(mg/L)	(g/L)		(mg/L)
c1	47	cluster center	30.75	138.42	24.39	121.24	13.28	257.68	0.59	7.78	0.85
		average	29.71	100.77	22.73	133.41	11.78	248.77	0.6	7.82	0.94
c2	16	cluster center	74.92	190.5	43.66	367.7	40.08	294.12	1.01	6.83	2.52
		average	81.26	172.53	40.25	360.55	46.76	271	0.97	6.72	2.33
c3	48	cluster center	156.67	220.02	80.15	597.63	38.46	266.32	1.36	7.51	3.65
		average	176.34	216.86	67.96	631.07	40.08	298.68	1.67	7.7	3.7
c4	33	cluster center	312.05	328.79	87.62	1224.59	88.61	294.39	2.4	7.42	1.33
		average	362.6	301.41	105.27	1166.16	137.91	325.44	2.45	7.35	1.34
	**Number of Samples**		${\mathrm{NO}}_{3}^{-}$	${\mathrm{NO}}_{2}^{-}$	${\mathrm{NH}}_{4}^{+}$	**Total Coliforms**	**Volatile Phenol**	**Cyanide**	**Cr^6+^**	**Pb**	**Zn**
			**(mg/L)**	**(mg/L)**	**(mg/L)**	**(/L)**	**(mg/L)**	**(mg/L)**	**(mg/L)**	**(mg/L)**	**(mg/L)**
c1	47	cluster center	1.96	0.01	0.02	0.99	0.001	0.008	0.008	0.002	1.26
		average	2.06	0.01	0.02	1.22	0.001	0.006	0.011	0.002	1.13
c2	16	cluster center	3.56	0.03	0.11	1.16	0.009	0.082	0.081	0.099	2.24
		average	3.42	0.02	0.08	1.17	0.008	0.071	0.09	0.087	2.54
c3	48	cluster center	5.04	0.03	0.07	2.42	0.002	0.04	0.031	0.052	1.9
		average	5.27	0.03	0.09	3.01	0.002	0.049	0.037	0.048	1.75
c4	33	cluster center	2.58	0.05	0.15	1.52	0.003	0.033	0.022	0.033	1.63
		average	3.41	0.07	0.17	1.38	0.002	0.025	0.026	0.029	1.78

**Table 6:** Pollutant concentration in factory sewage.

No.	Factory	Sewage Treatment and Discharge	${\mathrm{NO}}_{3}^{-}$	${\mathrm{NH}}_{4}^{+}$	COD_Mn_	Cr^6+^	Pb	Cyanide	Volatile Phenol	pH
			(mg/L)	(mg/L)	(mg/L)	(mg/L)	(mg/L)	(mg/L)	(mg/L)	
1	food factory	discharged directly into the ground	152.75	2.36	96.32	0.02	0.003	0.013	0.016	7.84
2	brickworks	discharged into river after simple treatment such as neutralization	186.2	4.55	158.67	0.017	0.012	0.034	0.037	7.05
3	electroplating factory	discharged into river after simple treatment such as neutralization and chemical precipitation	60.58	5.02	132.41	0.044	0.007	0.021	0.022	6.56
4	paper mill	discharged into river after simple treatment such as neutralization	34.01	15.89	141.6	0.035	0.019	0.082	0.025	6.81
average			108.39	6.96	0.038	0.025	132.25	0.046	0.01	7.07

**Table 7:** Concentrations of N pollutants in aquaculture ponds, drainage ditches, and offshore waters.

No.	Location	${NO}_{3}^{-}$	${NO}_{2}^{-}$	${NH}_{4}^{+}$	TN	pH
		(mg/L)	(mg/L)	(mg/L)	(mg/L)	
1	open-air sea cucumber pond	0.036	0.002	0.122	0.856	8.02
2	open-air shrimp pond	0.515	0.003	0.169	0.929	7.91
3	turbot pond in greenhouse	0.424	0.004	0.069	0.957	7.41
4	drainage ditch of open-sea cucumber pond	0.225	0.003	0.28	0.931	7.75
5	wastewater drainage of sea cucumber pond in greenhouse	0.31	0.003	0.393	1.664	7.86
6	drainage ditch of open-air shrimp pond	0.138	0.051	0.307	1.21	7.81
7	drainage ditch of pond in greenhouse	0.624	0.028	0.729	1.444	7.22
8	offshore water	0.013	0.005	0.062	0.457	7.15
9	offshore water	0.029	0.004	0.058	0.335	7.42
10	offshore water	0.021	0.004	0.046	0.461	7.33
average		0.234	0.011	0.224	0.924	7.59
